# Application of supervised and unsupervised machine learning approaches to identify somatic T‐cell receptor sequence variation in Alzheimer disease among Midwestern Amish

**DOI:** 10.1002/alz70855_102239

**Published:** 2025-12-23

**Authors:** Lauren A. Cruz, Shiying Liu, Kristy L. Miskimen, Yeunjoo E. Song, Renee A. Laux, Alan J. Lerner, Audrey Lynn, Sarada L. Fuzzell, Sherri D. Hochstetler, Ioanna Konidari, Jacob L. McCauley, William K. Scott, Margaret Pericak‐Vance, Jonathan L Haines, Dana C. Crawford

**Affiliations:** ^1^ Case Western Reserve University, Cleveland, OH, USA; ^2^ Cleveland Institute for Computational Biology, Case Western Reserve University, Cleveland, OH, USA; ^3^ Brain Health and Memory Center, University Hospital, Cleveland, OH, USA; ^4^ University of Miami, Miami, FL, USA; ^5^ John P. Hussman Institute for Human Genomics, University of Miami, Miami, FL, USA; ^6^ University of Miami Miller School of Medicine, Miami, FL, USA

## Abstract

**Background:**

Late‐onset Alzheimer disease (LOAD) is the most common form of dementia among adults ≥65 years of age. Known risk factors include female sex, increased age, mild cognitive impairment (MCI), and LOAD‐associated genetic risk alleles, most notably APOE e4. Recent evidence implicates the adaptive immune system as an important component of amyloid‐β plaque clearance and suggests, in limited sample sizes, LOAD‐associated immune profiles detectable in blood.

**Method:**

We characterized somatic T‐cell receptor (TCR) sequence diversity profiles from DNA extracted from blood (99%) and saliva (1%) in Ohio Amish participants with LOAD+MCI (*n* = 22), non‐LOAD (*n* = 35), and an unclear diagnosis (*n* = 12). Sequencing was performed using Adaptive Biotechnologies immunoSEQ targeting the beta chain, resulting in >160,000 productive templates and >100,000 productive rearrangements. TCR beta chain sequences were characterized using Simpson's productive clonality, which ranges from 0 to 1 representing diverse and completely even sequences (e.g., 0) to monoclonal or single dominant clone (e.g., 1). We used productive Simpson clonality as a measure of TCR diversity in our supervised logistic regression. To assess the pairwise repertoire similarities, we focused on the Morisita‐Horn index, which accounts for clonotype abundance while maintaining robustness against extreme values. We applied an unsupervised machine learning (ML) approach, fuzzy C‐means clustering, to identify potential patterns specific to disease status.

**Result:**

Participants did not differ significantly based on sex. LOAD+MCI participants were slightly older at last clinical exam than non‐LOAD or unclear participants (83.9 vs 81.5 and 81.2 years, respectively; chi‐square, *p* = 0.058). We observed significantly higher TCR sequence diversity in the non‐LOAD group compared to the LOAD‐MCI group (*p* = 0.027). However, the association was not significant after adjusting for age and sex. Clustering results showed moderate agreement with clinical diagnoses (adjusted rand index up to 0.4), and incorporating APOE genotypes enhanced the performance. The addition of age as a variable, which has been demonstrated to be related to both LOAD risk and TCR diversity, reduced the clustering agreement slightly.

**Conclusion:**

Decreased TCR diversity is marginally associated with having LOAD or MCI. Our ongoing work focuses on applying ML approaches to TCR CD3 amino acid sequences to identify disease‐associated clonotypes and develop predictive models for LOAD diagnosis.

